# Clinical Depression, Depressive Symptoms, and Depressed Cognition in Caregiving Grandmothers

**DOI:** 10.1093/geroni/igaa057.1186

**Published:** 2020-12-16

**Authors:** Christopher Burant, Alexandra Jeanblanc, Carol Musil, McKenzie Wallace, Zauszniewski Jaclene

**Affiliations:** 1 Case Western Reserve University, Parma, Ohio, United States; 2 Case Western Reserve University, Cleveland, Ohio, United States; 3 Case Western Reserve University, Lorain, Ohio, United States

## Abstract

Grandmothers living with or raising grandchildren have elevated levels of depressive symptoms compared to grandmothers who do not provide care. While the CES-D measures the somatic, positive and negative affect, and interpersonal strain symptoms experienced with depression, the Depressive Cognitions Scale captures the change in cognitive thinking that often precedes clinical depression. Our aim was to compare depressive symptoms and depressed cognitions between grandmother caregivers with a diagnosis of depression and those without in a nationwide sample of 342 grandmother caregivers. In the questionnaire, participants were asked whether they had a diagnosis of depression amongst other health conditions and also completed the CES-D and the Depressed Cognitions Scale. A score of 16 or greater on the CESD or a score above 7 on the Depressed Cognitions Scale can be used to identify individuals who may be at risk for depression. Grandmothers who had a diagnosis of depression were more likely to have CES-D scores 16 and above (79.7 %) as compared to those who were not (39.2%) (Chi Square=54.55, p<.001); and more likely to have higher depressed cognition scores 7 and above (71.3 %) as compared to those who did not (42.9%) (Chi Square=26.68, p<.001). Additionally, grandmothers who had depressed cognitions were more likely to have CES-D scores 16 and above (74.1 %) as compared to those who were not (33.1%) (Chi Square=57.56, p<.001). The elevated scores in participants who already have a diagnosis of depression indicates the need for potential interventions to further address depressive symptoms in grandmother caregivers.

